# Masked Image Modeling Meets Self-Distillation: A Transformer-Based Prostate Gland Segmentation Framework for Pathology Slides

**DOI:** 10.3390/cancers16233897

**Published:** 2024-11-21

**Authors:** Haoyue Zhang, Sushant Patkar, Rosina Lis, Maria J. Merino, Peter A. Pinto, Peter L. Choyke, Baris Turkbey, Stephanie Harmon

**Affiliations:** 1 Artificial Intelligence Resource, Molecular Imaging Branch, National Cancer Institute, Bethesda, MD 20814, USA; harry.zhang@nih.gov (H.Z.); sushant.patkar@nih.gov (S.P.); rosina.lis@nih.gov (R.L.); pchoyke@nih.gov (P.L.C.); ismail.turkbey@nih.gov (B.T.); 2 Translational Surgical Pathology Section, National Cancer Institute, National Institutes of Health, Bethesda, MD 20814, USA; mjmerino@mail.nih.gov; 3 Urologic Oncology Branch, National Cancer Institute, National Institutes of Health, Bethesda, MD 20814, USA; pintop@mail.nih.gov

**Keywords:** digital pathology, whole slide image, Deep Learning, gland segmentation, prostate cancer

## Abstract

Detailed evaluation of prostate cancer glands is an essential yet labor-intensive step in grading prostate cancer. Gland segmentation can serve as a valuable preliminary step for machine-learning-based downstream tasks, such as Gleason grading, patient classification, cancer biomarker building, and survival analysis. Despite its importance, there is currently a lack of a reliable gland segmentation model for prostate cancer. Without accurate gland segmentation, researchers rely on cell-level or human-annotated regions of interest for pathomic and deep feature extraction. This approach is sub-optimal, as the extracted features are not explicitly tailored to gland information. Although foundational segmentation models have gained a lot of interest, we demonstrated the limitations of this approach. This work proposes a prostate gland segmentation framework that utilizes a dual-path Swin Transformer UNet structure and leverages Masked Image Modeling for large-scale self-supervised pretaining. A tumor-guided self-distillation step further fused the binary tumor labels of each patch to the encoder to ensure the encoders are suitable for the gland segmentation step. We united heterogeneous data sources for self-supervised training, including biopsy and surgical specimens, to reflect the diversity of benign and cancerous pathology features. We evaluated the segmentation performance on two publicly available prostate cancer datasets. We achieved state-of-the-art segmentation performance with a test mDice of 0.947 on the PANDA dataset and a test mDice of 0.664 on the SICAPv2 dataset.

## Simple Summary

The accurate characterization of prostate cancer glands is vital for diagnosis and grading, and the segmentation of these glands is critical for quantitative analysis and machine learning models. Human annotation is unrealistic given the size of whole-slide images, yet there lacks a reliable fully automated segmentation framework. This study addressed the limitations of previous state-of-the-art approaches reported on this task, popular segmentation frameworks, and foundational models. Our proposed framework leverages the strength of self-supervised and self-distillation to achieve a high segmentation performance, offering a reliable prostate gland segmentation to pave the way for better downstream analysis of whole-slide images.

## 1. Introduction

Prostate cancer (PCa) diagnosis and grading rely on an analysis of whole-slide pathology images (WSIs) [1]. Pathological assessment via the Gleason grading system is the foundation of risk assessment and prognostication in localized PCa [2,3]. Within the Gleason grading system, the scoring criteria that pathologists use to identify and distinguish the severity of disease are based on the morphology and growth patterns of prostate cancer glands [2,4]. PCa is known to exhibit a high level of heterogeneity in the appearance and complexity of various histomorphological architectures, and suffers from poor inter-observer agreement [5,6]. Inter-reader agreement worsens as the glandular architecture becomes more complex, which occurs as cancerous glands diverge away from low-risk well-formed glands (Gleason 3) to higher-risk patterns of poorly formed/fused glands (Gleason 4) [6].

More recently, multiple AI-based reports demonstrate that automated algorithms can match or exceed the performance of pathologists for detection and grading [7]. Despite the underlying dependency on glandular shape and distribution on defining Gleason grades, the vast majority of AI-based approaches for the automated detection and grading of prostate cancer rely on multiple instance-learning-based algorithms trained from weakly supervised slide-level or whole-tumor-level labels [8,9,10,11,12,13,14], and very few utilize segmentation strategies [7,15,16,17]. Given the large size of digital WSIs, these approaches are typically constructed from tile-based approaches where each image tile is labeled by the slide-level diagnosis. However, training from weakly labeled data does not explicitly account for the critical features of architectural patterns that enable important downstream tasks, such as detection and accurate quantification of the tumor burden [18,19], assessment and distribution of glandular morphology [7,17], and identifying interpretable patterns of high-risk disease [20]. Furthermore, these multiple instance learning (MIL) approaches may over-fit the source of tissue data, i.e., tissue micro-array core, biopsy core, or surgical specimens, where weak labels variably capture the extent and heterogeneity of the disease. Therefore, we argue that an algorithmic approach for the automated segmentation of glandular structures of prostate cancer is a critical, foundational task for further detection, grading, and prognostication tasks.

One challenge when developing gland-level segmentation algorithms is the lack of high-quality annotated data for individual cancer glands due to the complexity and heterogeneity of the disease, in addition to the time-consuming nature of this manual task for experts to generate data for training. Segmentation on WSIs remains a challenging task. Large foundational visual models have recently shown promising zero-shot segmentation capabilities in natural images [21]. Medical or pathology-focused approaches demonstrated state-of-the-art performance across different tasks [22,23]. However, based on our experiments, while the zero-shot performance of the SAM and MedSAM is impressive, fine-tuning these models does not yield higher performance. Therefore, a customized model tailored to this challenging prostate gland segmentation task is still worth exploring.

In this work, we leveraged a dual-path multi-scale cross-attention network based on a Swin Transformer architecture inspired by [24] and propose several modifications to improve the model’s efficiency. We employed Masked Image Modeling to leverage the power of large-scale unlabeled domain-specific data to pretrain two Swin Transformer encoders. We propose a tumor-guided self-distillation process to fuse in the patch-level label to adjust the features for gland segmentation training. Finally, our segmentation training contained two steps. First, we used the PANDA Challenge dataset [25] to train the gland segmentation task by leveraging the PANDA dataset’s massive yet incomplete and unreliable pixel-level annotations, and then fine-tuned this on the SICAPv2 dataset [26], which is small but with higher-quality pixel-level annotation. The experiments showed that the proposed gland segmentation framework achieved state-of-the-art performance on open-source datasets, where it out-performed nnUNet and two major large foundational models. Our main contributions can be summarized as follows:

- We propose a simple framework that bridges the self-supervised Masked Image Modeling pertaining and prostate gland segmentation tasks by a tumor-guided self-distillation step.
- We evaluated major segmentation methods, including large foundational SAMs, and demonstrated the limitation of the large foundational model and the value of the specially tailored model in histopathology slides.
- We curated a large-scale whole-mount dataset with patch-level tumor labels for the research community to enhance their self-supervised learning models.

## 2. Materials and Methods

### 2.1. Dataset

**Figure 1:**
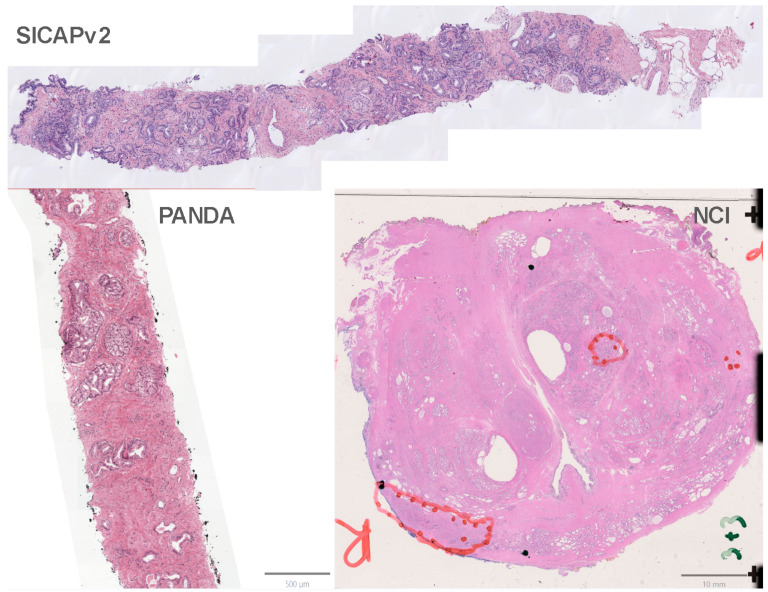
Sample slides from the three data cohorts. The top slide is from SICAPv2. Note that the SICAPv2 dataset is provided in a patch form, so the sample shown in this figure was stitched back based on the given coordinates. The bottom-left slide is from the PANDA cohort. The bottom-right slide is a whole-mount slide from our in-house dataset NCI.

Our study utilized three datasets for prostate gland segmentation Figure 1. Some data provided patch-level tumor labels. (1) SICAPv2 is a public dataset that comprises 155 WSI biopsy slides from 95 patients. It provides 18,783 patches that each measure 512 × 512 pixels and are extracted from whole-slide images captured at 40× magnification and then down-sampled to 10× magnification. The tiles were annotated by expert pathologists at the pixel-level to generate the segmentation masks with Gleason grades. (2) The PANDA Challenge is a large-scale public dataset from the Radboud and Karolinska cohorts. The Radboud dataset includes 5759 prostate biopsies from 1243 patients at Radboud University, Netherlands. The slides were captured at 20× magnification and downsampled to 10×. The Radboud dataset provides noisy pixel-level Gleason pattern masks. The Karolinska dataset contains 5662 core needle biopsies from 1222 patients from various hospitals in Sweden. We extracted 547,386 and 822,082 512 × 512 patches from Radboud and Karolinska, respectively. (3) An in-house dataset NCI includes 150 whole-mount slides with patch-level tumor labels. The slides were digitized at 40× magnification and down-sampled to 10×, resulting in 273,405 512 × 512 patches extracted.

### 2.2. Preprocessing

Given that the SICAPv2 dataset provides data as 512 × 512 patches at 10×, we aimed to process the WSIs from two cohorts in a similar fashion, generating tissue patches from PANDA biopsy samples and dataset NCI whole-mount surgical specimens at the same size and resolution as SICAPv2. For the PANDA Challenge dataset, we processed both the Radbound and Karolinska cohorts using CLAM [27], a fully automated pipeline that segments tissue contents and removes holes to generate tissue patches efficiently. For the CLAM patch creation process, we used the following parameters: segmentation threshold sthresh = 15, median filter size mthresh = 11, and additional morphological closing after initial thresholding close = 2, and we used Otsu’s method for thresholding. For the contour-filtering parameters, we used the area filter threshold for tissue a_t = 1, area filter threshold for holes a_h = 1, and the maximum number of holes to consider per detected foreground contour max_n_holes = 2; the rest of the settings followed the default bwh_biopsy.csv file provided by the author. After processing, 200 cases were randomly picked and visually checked for quality assurance using the low-resolution stitched image. For the NCI dataset, we used in-house code based on the bioformats Python library, which segments the tissue before patch extraction. We did not filter to cancer-specific regions in the surgical specimens, which resulted in the majority of patches reflecting the heterogeneous distribution of non-cancerous pathology features in the entire prostate organ.

### 2.3. Masked Image Modeling

To leverage the large-scale public and in-house dataset with unlabeled data, generative self-supervised learning (SSL) was employed. Contrastive-based SSL or Masked Image Modeling (MIM) has attracted much attention in recent studies. Although contrastive SSL shows excellent feature extraction and zero-shot capabilities, it falls short for downstream fine-tuning compared with MIM [28,29]. Since SSL is only our first step in the training pipeline, we employed the SimMIM approach [30] for its superior performance with the Swin Transformer architecture. Swin-Tiny and Swin-Base encoders were trained separately by SimMIM for the tumor-guided self-distillation step. Briefly, SimMIM utilizes the same strategy as a Masked Autoencoder (MAE) [31], which masks a random portion of image signals and predicts the original signals in the masked area through the reconstruction process. Unlike MAE, SimMIM only uses pixel-shuffling and one linear layer as a highly lightweight decoder. The loss function is described as

(1) $$L_{l1}=\frac{1}{n}\sum_{i=1}^{n}\left|y_{i}-{\hat{y}}_{i}\right|$$

where $y_{i}$ is the predicted pixel and ${\hat{y}}_{i}$ is the masked true pixel.

### 2.4. Tumor-Guided Self Distillation

Directly fine-tuning from the SimMIM pre-trained model showed limited improvement. This could have been due to the gap of both deep and shallow features between reconstruction and segmentation. We propose a simple yet effective feature-based deep self-distillation step to address this issue. Instead of distilling logits at the end of the teacher and student model, inspired by deep supervision [32], we used features at different stages as supervision, where the Swin Transformer encoder could be divided into four stages given the original design. Patch-level tumor/no-tumor labels are usually easier to obtain than pixel-level annotations. A naive way to use this binary label would be to train the encoder on a patch-level classification task. However, given that our goal was prostate gland segmentation, an encoder directly trained for such a task may compromise the features of low-level tasks. Instead, we propose using patch-level tumor/no-tumor labels as hard attention to fuse into the features at the multi-scale stages of the Swin Transformer encoder during the self-distillation process. Formally, given a teacher model $M_{t}$ and a student model $M_{s}$, we have $F_{t}^{i}$ and $F_{s}^{i}$, where i=1,2,3,4 corresponds to the feature maps at the end of each stage of the Swin Transformer. The binary label *G* is reshaped to the same size as $F_{t}^{i}$ and fused through teacher model $M_{t}$’s features $F_{t}^{i}=G_{r}eshape⨂w_{i}+F_{t}^{i}$, where $w_{i}$ is the weighted factors for each stage. The features from the student model $M_{s}$ pass through a bridge module $B_{s}=Conv1D+LayerNorm$ before calculating the loss. The loss is defined as

(2) $$\mathrm{Final}\;\mathrm{Loss}=\frac{1}{N}\sum L_{1;\mathrm{smooth}}\left(F_{t}^{i},B_{s}\left(F_{s}^{i}\right)\right)$$

where N was 4 in this study.

### 2.5. Prostate Gland Segmentation

**Figure 2:**
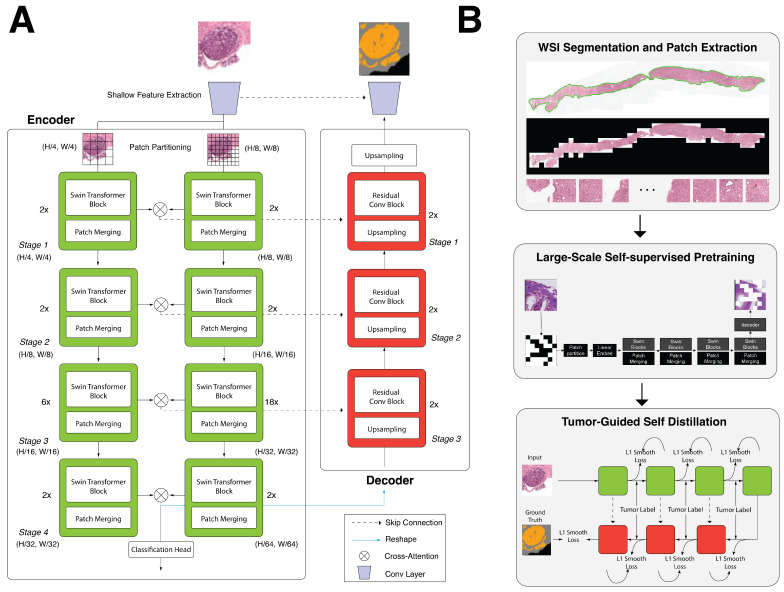
Overview of the proposed model for prostate gland segmentation. Section (**A**) shows the architecture of our proposed dual-path segmentation architecture. Section (**B**) shows our preprocessing, self-supervised learning, and self-distillation schema for the self-supervised learning step.

The overall architecture of the proposed dual-path Swin Transformer segmentation model is shown in Figure 2A. We built the UNet structure inspired by [24] and made several improvements (Figure 2B). First, we introduced a shallow feature extractor from [33,34,35] as the first step. Similar to that shown in a study by Xiao et al. [36], we observed improved convergence speed and more stable training. The shallow feature extractor helps capture low-frequency information [33] while providing simple mappings from the input image space to a higher-dimensional feature space to reduce optimization difficulties. Afterward, the output splits into two branches of the Swin Transformer encoder with different patch sizes, 4 and 8, to capture coarse and fine features at different spatial levels. At the end of each Swin Transformer stage, the features from two branches are fused. Ref. [24] provides a complex interactive operation module, which introduces a lot of computational costs. We simplified the interactive module to the following form: Given two features $F_{a}^{i}$ and $F_{b}^{i}$ from the same stage *i* for the two branches, the features first go through

(3) $$F_{a/b}^{i}=FeedForward\left(AvgPool\left(LayerNorm\left(F_{a/b}^{i}\right)\right)\right)$$

Then, the features are generated by

(4) $$F_{fused}=Conv\left(Attention\left(Concat\left(F_{a}^{i},F_{b}^{i}\right)\right)\right)$$

where a multi-head attention module is used to focus on important features from both branches and a convolution layer is used to reshape back to the original feature size of $F_{a}^{i}$ for a more straightforward decoding operation. Instead of pursuing the sole usage of the Swin Transformer blocks for decoder, we used the same bottleneck operations, deconvolutional layers, and residual connections as in UNETR [37]. This decoder is less computationally intensive yet has no adverse impact on the performance. At last, we added a classification head for each of the two encoders to determine whether the input patch contained tumors. We used the average of the two classification heads’ logits to make the prediction and force the model to output a negative segmentation mask only when the confidence of the classification is above 90% to call it a non-tumor patch. This strategy effectively removed some small segmentation outputs that should not appear on a negative patch. The loss function combines weighted IoU loss, binary focal loss with deep supervision at the same location described in [24], and binary focal loss in the classification head.

(5) $$\begin{array}{cc} \mathrm{Final}\;\mathrm{Loss} & =0.6L\left(G,S1\right)+0.2L\left(G,S2\right)+0.2L\left(G,S3\right)+L_{focal}\\ L(G,S_{x=1,2,3}) & =L_{IoU}^{W}+L_{BCE_{focal}} \end{array}$$

where G is the ground truth pixel and Sx represents the outputs at different stages of the Swin Transformer encoder. $L_{IoU}^{W}$ represents the weighted IoU loss, $L_{BCE_{focal}}$ represents the binary focal loss at each stage, and $L_{focal}$ represents the binary focal loss of the classification head.

### 2.6. Model Training and Evaluation

Each step is summarized in Table 1 with corresponding models trained and datasets used. All patches from the three data sources (more than 1.6 million 512 × 512 patches in total), except the hold-out test set, were first trained through SimMIM self-supervised learning for 200 epochs for both Swin-Tiny and Swin-Base encoder setups with patch sizes 8 and 4. In the second step, patches with patch-level binary tumor labels (1 million patches) were used in the tumor-guided self-distillation process.

The SICAPv2 and PANDA datasets were utilized to train the prostate gland segmentation model. Extensive data augmentations were applied. Random 90 degrees rotation and random horizontal and vertical flips were applied with 0.1 probability; one of the following transforms was called during each iteration with a probability of 0.1: Gaussian smoothing with sigma 0.1 to 1.1; median smoothing with radius 1; Gaussian noise with a standard deviation of 0.05; and color jittering with brightness from 229 to 281, contrast 0.95 to 1.1, saturation 0.8 to 1.2, and hue −0.04 to 0.04 were applied afterward. After all augmentations, the RGB values were scaled to the 0 to 1 range. Training on PANDA–Radboud only served as an intermediate step for the prostate gland segmentation, given the relatively low reliability of the provided segmentation mask. In the final step, we fine-tuned the SICAPv2 dataset to achieve the best performance. We used the SICAPv2-provided training, validation, and test split, accounting for the data distribution and label ratios. PANDA–Radboud was randomly split into 70%, 10%, and 20% for training, validation, and testing. The SGD optimizer with a momentum of 0.9, weight decay of 0.0001, and a learning rate of 0.005 was used for training from scratch, and a learning rate of 0.0001 for our model’s fine-tuning stage or SAM/MEDSAM fine-tuning. A DGX2 with 8 A100 40 GB (NVIDIA, Santa Clara, CA, USA) was used for training.

The mean Dice Similarity Coefficient (mDice) and mean Intersection over Union (mIoU) were used for the performance comparison throughout the experiments. Each model output pixel can be categorized into a True Positive (TP), False Positive (FP), True Negative (TN), or False Negative (FN). Given the four categories, the mean Dice and mean IOU can be computed using the following formulas:

(6) $$\mathrm{mDice}=\frac{2\times TP}{2\times TP+FP+FN}$$

(7) $$\mathrm{mIoU}=\frac{TP}{TP+FP+FN}$$

## 3. Results and Discussion

To evaluate the effectiveness of our proposed method, we conducted an ablation study by comparing the performances of the different model variants presented in Table 2. Specifically, the base model was the proposed dual-path Swin UNet trained from scratch. We systematically added one or more training strategies to the base model to evaluate their performance contributions. The results show that the full model outperformed the base model by large margins of 4.5% in the mean Dice and 5% in the IOU, and each training step contributed to the overall performance. Directly adding self-supervised MIM training with the two public datasets and the in-house dataset showed a steady performance gain, but the largest improvement in the performance was from self-distillation. Note that for the self-distillation step, we used all the available datasets, including the in-house NCI dataset that contained the patch-level tumor/no-tumor labels. Fusing this discriminative information into every stage of the encoder provided better feature representation for the segmentation task, which explained the effectiveness of the self-distillation step.

The performance for PANDA training achieved a test mean Dice of 0.947 and a mean IOU of 0.974. However, due to the unreliable quality of the PANDA segmentation masks, we decided not to report the results in the table. However, the training was still valuable and provided good generalizability for a large cohort, as we directly tested the PANDA-trained model on SICAPv2 and achieved a mean Dice of 0.664 and a mean IOU of 0.550, which showed a higher zero-shot capability than the two foundational models SAM and MedSAM.

**Figure 3:**
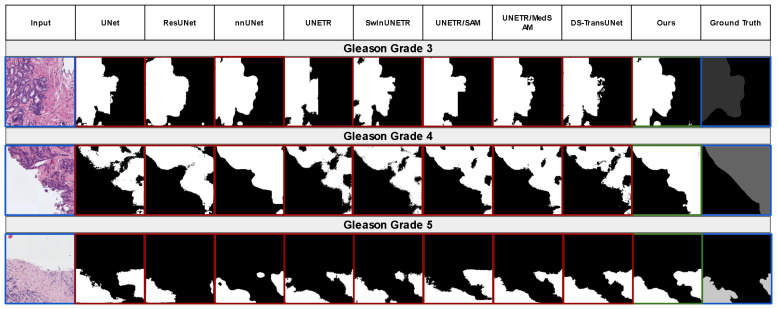
Sample segmentation results for different Gleason grade glands across different methods. Compared with other methods, many small spots were removed by the tumor classification head in our network, which yielded a better visual representation without any post-processing smoothing methods.

As shown in Table 3, we systematically evaluated the baseline approaches and several major works. Sample segmentation results are also shown for qualitative comparison in Figure 3. All models were trained from scratch for a fair comparison, except for UNETR + SAM and UNETR + MedSAM, where we examined whether we could achieve state-of-the-art performance through fine-tuning these two large-scale foundational segmentation models’ encoders [21,22]. Our initial work started by trying to train a model tailored to prostate gland segmentation from the SAM or MedSAM to serve as a first step for downstream tasks in prostate cancer analysis. Although the SAM and MedSAM showed promising zero-shot performances out-of-the-box in our evaluation (mean test Dice scores of 0.55 and 0.56 on SICAPv2, respectively), further fine-tuning did not yield state-of-the-art performance. These results inspired us to explore a more customized training approach for prostate gland segmentation.

The baseline methods, such as UNet and ResUNet, achieved similar performances. SwinUNETR outperformed UNETR, and DS-TransUNet performed similarly to UNETR + MedSAM. Some methods had higher validation mean Dice scores but lower test mean Dice scores; this could have been because the SICAPv2 official data split was stratified on patient-level information, not on the distribution of annotation masks. Both the validation and test mean Dice scores are reported for transparency purposes. Based on our extensive experiments, the test mean Dice and IOU were aligned with the training loss and validation loss, and given we chose minimal validation loss as the selection criteria for the best epoch for testing, the validation mean Dice did not change the implications of this study. As mentioned above, although using the SAM or MedSAM pre-trained weights only yielded around a 5% increase over the UNETR trained from scratch, their zero-shot performances were higher than many of the baseline methods. One possible explanation for the SAMs not reaching a higher performance could have been the model’s weights being affected by too much data trained from other scenarios. Fine-tuning on a smaller domain-specific dataset may require specifically designed training strategies. Research on reliable fine-tuning strategies for large foundational models is needed. Potential future directions could be around domain adaptations or progressive fine-tuning for the SAMs. On the other hand, segmentation on the SICAPv2 dataset is a very challenging task. The previous state-of-the-art approaches SegGINI and the following work [10,42] attempted to segment the gland and achieved a relatively low performance. Although their optimization goal of the Gleason grade was more challenging, this was partially because the high-grade Gleason grade ground truth is less consistent. Nevertheless, their average Dice of 0.476 can be directly compared with our result, with an 18.8% performance difference. Recently, [23] attempted to build a large visual-language foundation model in digital pathology and evaluated their performance on SICAPv2 for gland segmentation, and their proposed method achieved a 0.601 mean Dice. Our approach exceeded the performances of these studies that attempted the same or similar segmentation task. There were a few limitations in this study. Even though more than 18,000 patches exist in the SICAPv2 datasets, they were only from a single center. Semi-automated expert annotation should be investigated to improve the ground truth on PANDA datasets, our in-house NCI dataset, and other public prostate WSI datasets. Although the quality of the SICAPv2 annotation is high compared with other public datasets, it still neglects many fine details. During the evaluation stage, we randomly picked 50 patches from SICAPv2 for two expert pathologists to review the ground truth and the segmentation results. Both pathologists agreed that the SICAPv2’s ground truth masks are relatively forgiving at the contours of the glands, which the segmentation models can differentiate quite well. Therefore, the outputs of all the models that are over-segmented in fine detail may not necessarily mean they are wrong.

## 4. Conclusions

In conclusion, we propose a novel training framework for prostate gland segmentation on WSIs, incorporating large-scale Masked Image Modeling pretraining and self-distillation. Moreover, our segmentation model leverages the features at different spatial scales through a dual-path Swin Transformer UNet structure. The SICAPv2 and PANDA Challenge results suggest our proposed framework achieved state-of-the-art WSI segmentation performance.

## Figures and Tables

**Table 1:** Training steps and the associated datasets.

Training Step	Models Trained	Datasets Used
SimMIM self-supervised	Swin-Tiny and Swin-Base encoders	PANDA, SICAPv2, NCI
Tumor-guided self-distillation	Swin-Tiny and Swin-Base encoders	PANDA, SICAPv2, NCI
Segmentation step 1	Segmentation encoders and decoders	PANDA
Segmentation step 2	Segmentation encoders and decoders	SICAPv2

**Table 2:** Ablation study comparison with the model variants. Results are given as the mean Dice and mean IOU for validation and test sets with a 95% bootstrap confidence interval at the slide level. The base was our proposed dual-path Swin UNet architecture trained from scratch. The results were based on the SICAPv2 dataset.

Methods	Val mDice	Val mIoU	Test mDice	Test mIoU
Base	0.728 (0.698, 0.752)	0.631 (0.602, 0.647)	0.619 (0.599, 0.637)	0.500 (0.477, 0.523)
+MIM two public datasets	0.732 (0.715, 0.749)	0.638 (0.615, 0.658)	0.622 (0.600, 0.642)	0.505 (0.488, 0.524)
+MIM in-house dataset	0.738 (0.723, 0.746)	0.645 (0.632, 0.655)	0.625 (0.614, 0.635)	0.512 (0.500, 0.527)
+Self-distillation	0.754 (0.737, 0.771)	0.662 (0.644, 0.681)	0.641 (0.625, 0.656)	0.521 (0.509, 0.536)
+PANDA dataset training	**0.782 (0.771, 0.795)**	**0.697 (0.685, 0.707)**	**0.664 (0.651, 0.677)**	**0.550 (0.538, 0.561)**

**Table 3:** Gland segmentation comparison on the SICAPv2 dataset. All methods were trained from scratch for a fair comparison, except for the SAM and MedSAM, which were fine-tuned to the best performance. Our proposed methods are shown as trained from scratch vs. the final results, as shown in the ablation study, for easier comparison.

Method	Val mDice	Val mIoU	Test mDice	Test mIoU
UNet [38]	0.612 (0.594, 0.629)	0.511 (0.493, 0.529)	0.480 (0.464, 0.496)	0.363 (0.347, 0.380)
ResUNet [39]	0.605 (0.586, 0.623)	0.493 (0.473, 0.511)	0.534 (0.511, 0.545)	0.424 (0.406, 0.434)
UNETR [37]	0.684 (0.670, 0.697)	0.585 (0.571, 0.599)	0.531 (0.518, 0.545)	0.416 (0.402, 0.429)
UNETR+SAM [21]	0.653 (0.642, 0.668)	0.555 (0.542, 0.568)	0.574 (0.569, 0.590)	0.458 (0.447, 0.475)
UNETR+MedSAM [22]	0.679 (0.668, 0.690)	0.581 (0.571, 0.592)	0.584 (0.571, 0.598)	0.471 (0.456, 0.485)
SwinUNETR [40]	0.705 (0.693, 0.718)	0.606 (0.593, 0.619)	0.563 (0.547, 0.576)	0.440 (0.429, 0.458)
DS-TransUNet [24]	0.702 (0.691, 0.712)	0.605 (0.594, 0.616)	0.583 (0.570, 0.602)	0.471 (0.460, 0.490)
nnUNet v2 [41]	0.703 (0.694, 0.713)	0.609 (0.600, 0.618)	0.532 (0.523, 0.542)	0.439 (0.429, 0.448)
Ours (scratch)	0.728 (0.698, 0.752)	0.631 (0.602, 0.647)	0.619 (0.599, 0.637)	0.500 (0.477, 0.523)
Ours (final)	0.782 (0.771, 0.795)	0.697 (0.685, 0.707)	0.664 (0.651, 0.677)	0.550 (0.538, 0.561)

## Data Availability

The internal datasets generated during and/or analyzed during the current study are available from the corresponding author upon reasonable request.
